# What can we conclude about the effect of parental income on offspring mental health?

**DOI:** 10.1093/ije/dyac144

**Published:** 2022-07-06

**Authors:** Guido Biele, Johan de Aguas, Tomás Varnet Pérez

**Affiliations:** Department of Child Health and Development, Norwegian Institute of Public Health, Oslo, Norway; Department of Child Health and Development, Norwegian Institute of Public Health, Oslo, Norway; Department of Child Health and Development, Norwegian Institute of Public Health, Oslo, Norway

Two articles recently published in the *International Journal of Epidemiology* came to different conclusions about the association between parental income and offspring’s mental health diagnoses. Using panel data analysed with logistic regression and adjustment for observed confounders, Kinge *et al.*^1^ reported a negative association in Norwegian data. Sariaslan *et al.*^2^ implemented a discordant siblings design study and stratified Cox regression to reduce bias from unmeasured confounders (but also see Frisell *et al*.^3^) and concluded that there is no causal association in Finnish data. The two articles describe their results in associative vs. causal language, but both studies ultimately report associations whose causal interpretation depends on untestable assumptions, and associations from both studies can be seen as potentially biased estimators of the causal effect in target population.

To investigate if the diverging results are due to different study populations, study designs, statistical approaches or definitions of exposure and outcome, we analysed data from Norwegian children with Scandinavian parents born between 1997 and 2012. We implemented the analytical approaches used in the two articles and a conditional logistic regression model^4^ that investigates effects of year-to-year income variation while controlling for unobserved unit-level heterogeneity. We used income percentiles as exposure, and diagnoses registered in the National Patient Register from child age 5 years on until child age 21 or the year 2017 as outcomes. The sample included 557 056 children with a prevalence of 4.8%, 2.1% and 4.6% for attention-deficit hyperactivity disorder (ADHD), depression and anxiety/compulsive obsessive disorders, respectively. Due to the requirement of outcome-discordant individuals within strata, sample sizes for some analyses are considerably smaller, with ∼16 000 to 35 000 for the sibling design and ∼12 000 to 26 000 for the panel study design.

The left panel in Figure 1 shows that, replicating the results of Kinge *et al*., higher parental income is associated with lower risk for mental health diagnoses. This association is stronger among children whose parents do not have a mental health diagnosis. The middle panel shows that accounting for unit-level fixed effects with a conditional logistic model substantially weakens the income-mental health associations. The third panel shows that, replicating the results of Sariaslan *et al*., employing a sibling design results mostly in associations that no longer reach statistical significance. These findings show that, using the same dataset and exposure definition, different assumptions and resulting analytical approaches lead to results that do or do not support a causal association between parental income and child mental health.^2^

**Figure 1:**
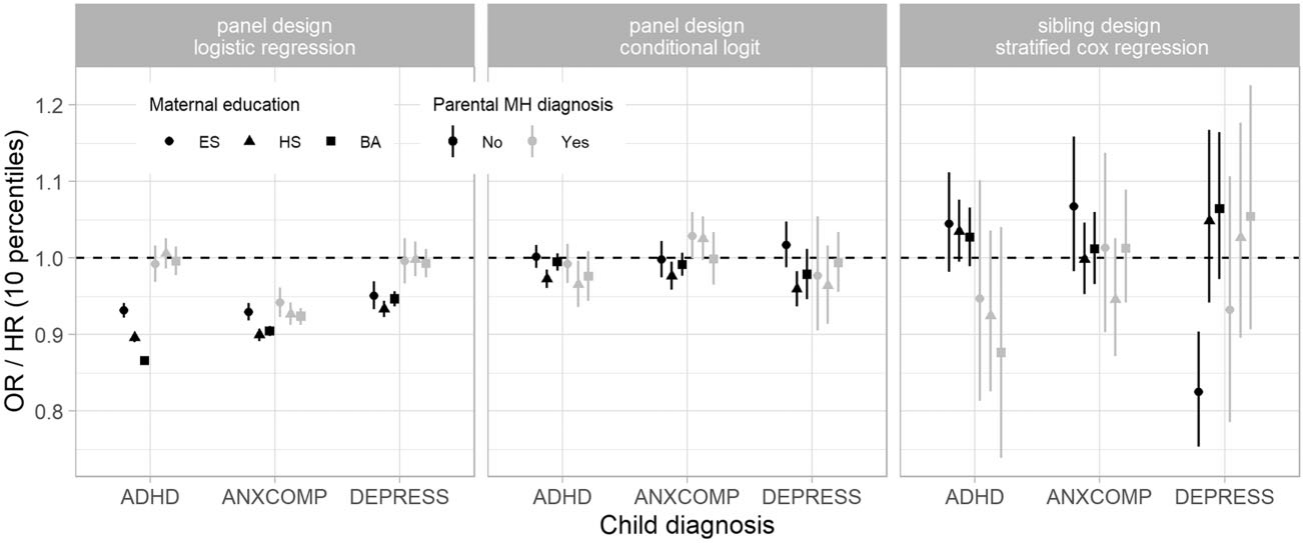
Estimated association measures between parental income and child mental health diagnoses from different study designs and statistical approaches. Vertical lines indicate 95% confidence intervals calculated from clustered standard errors. The panel design with logistic regression captures associations due to variation of parental income within and between children, the conditional logistic analysis associations due to variation within children, and the sibling design associations due to variation between siblings. MH, mental health; ES, elementary school (10 years); HS, high school (13 years); BA, Bachelor or higher education (more than 15 years); ADHD, attention-deficit hyperactivity disorder; ANXCOMP, anxiety or obsessive compulsive disorders; DEPRESS, depression; OR, odds ratio; HR, hazard ratio

Given the more obvious problem of unobserved confounding in the analysis of Kinge *et al.*, some prefer estimates from sibling or panel design studies as estimates of a causal effect. However, such studies use only a small, specific subset of the target population, in which the variation of the exposure is substantially smaller than in the population (compare Ledberg e*t al.*^5^). We found that within-strata variation of income accounts for only about 23% of the total income variation, and that within-strata variability is characterized by mostly very small changes.

In addition to previously described biases specific to sibling design studies,^3^^,^^5^^,^^6^ sibling design studies are at risk for selection bias. To include discordant outcomes within strata, they have to select participants based on the outcome. If income is the exposure and, as is the case in European countries, higher income is associated with higher likelihood for a second child,^7^ selection will depend on exposure and outcome, thus creating the potential for selection bias due to conditioning on a collider.^8^ Because the positive effect of parental income and child diagnosis on study participation results in an upwards bias for the estimate of the income-mental health association, a true negative effect of parental income will be attenuated in a sibling design study sample. Figure 2 illustrates a simulation that shows how selection bias in sibling design studies can hide a true causal effect of parental income on children’s mental health.

**Figure 2:**
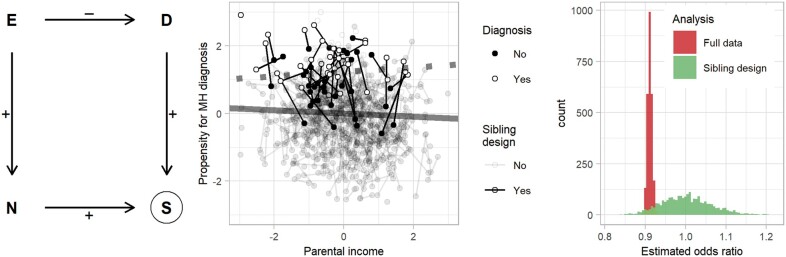
Selection bias in sibling designs. Left: directed acyclic graph for selection bias in a sibling design study with binary outcomes. Collider bias manifests if the exposure is also an (indirect) cause of participation, because study participation depends on the outcome being present in at least one child. Centre: we simulated data with a true odds ratio of .91 for the effect of the exposure (income) on the outcome in the population (prevalence 6%). Consistent with Norwegian data, the exposure is assumed to be highly correlated between siblings (r = 0.9), a 1-standard deviation increase of income is assumed to increase the chance for a second child by 10%, and siblings’ mental health is assumed to be correlated (r = 0.7). The dashed bold line shows the estimated association for the population and the solid line for sibling design data, for 500 families from a simulated dataset. Right: effect estimates from 2500 simulated populations, each with 300 000 families. MH, mental health

The articles of Kinge *et al.* and Sariaslan *et al.* provide valuable insight into the relationship between parental income and children’s mental health, and at the same time highlight the strong dependency of study results on implicit and explicit assumptions. Study designs that reduce one type of bias can simultaneously increase risk for another bias, and choosing between designs can mean trading off one type of bias for another. In a triangulation approach, similar results from studies with different biases can provide evidence supporting a causal effect of the exposure on the outcome.^9^ In the articles discussed here, results from studies with different biases are inconsistent; so that considerable uncertainty about a causal effect of parental income on children’s mental health remains. One potential approach to reduce this uncertainty is to use a triangulation approach with more study types with other biases, including instrumental variable approaches like Mendelian randomization^10^ or natural experiments.^11^

## Ethics approval

The research was approved by the Regional Comittees for Medical Research Ethics South East, project number 26684. The study was conducted in compliance with international standards such as the Declaration of Helsinki.

## Data Availability

The relevant data can be obtained from Statistics Norway [https://www.ssb.no/en/data-til-forskning] and the Norwegian Patient Registry [https://helsedata.no/en/forvaltere/norwegian-directorate-of-health/norwegian-patient-registry-npr/].
